# BH_4_-deficient hyperphenylalaninemia in Russia

**DOI:** 10.1371/journal.pone.0249608

**Published:** 2021-04-06

**Authors:** Polina Gundorova, Irina A. Kuznetcova, Galina V. Baydakova, Anna A. Stepanova, Yulia S. Itkis, Victoria S. Kakaulina, Irina P. Alferova, Lidya V. Lyazina, Lilya P. Andreeva, Ilya Kanivets, Ekaterina Y. Zakharova, Sergey I. Kutsev, Aleksander V. Polyakov

**Affiliations:** 1 Research Centre for Medical Genetics, Moscow, Russia; 2 Federal State Budgetary Institution of Medical Department of Moscow “Morozov Children’s City Clinical Hospital of Medical Department of Moscow”, Moscow, Russia; 3 Municipal Autonomous Health Care Institution of the Order of the Red Banner of Labor “Clinical Hospital № 1”, Chelyabinsk, Russia; 4 Saint Petersburg State Public Health Institution "Medical Genetic Diagnostic Center”, Saint Petersburg, Russia; 5 Saratov Regional Children’s Clinical Hospital, Saratov, Russia; 6 Genomed, Moscow, Russia; Universita degli Studi di Roma Tor Vergata, ITALY

## Abstract

A timely detection of patients with tetrahydrobiopterin (BH_4_) -deficient types of hyperphenylalaninemia (HPABH_4_) is important for assignment of correct therapy, allowing to avoid complications. Often HPABH_4_ patients receive the same therapy as phenylalanine hydroxylase (PAH) -deficiency (phenylketonuria) patients—dietary treatment—and do not receive substitutive BH_4_ therapy until the diagnosis is confirmed by molecular genetic means. In this study, we present a cohort of 30 Russian patients with HPABH_4_ with detected variants in genes causing different types of HPA. Family diagnostics and biochemical urinary pterin spectrum analyses were carried out. HPABH_4_A is shown to be the prevalent type, 83.3% of all HPABH_4_ cases. The mutation spectrum for the *PTS* gene was defined, the most common variants in Russia were p.Thr106Met—32%, p.Asn72Lys—20%, p.Arg9His—8%, p.Ser32Gly—6%. We also detected 7 novel *PTS* variants and 3 novel *QDPR* variants. HPABH_4_ prevalence was estimated to be 0.5–0.9% of all HPA cases in Russia, which is significantly lower than in European countries on average, China, and Saudi Arabia. The results of this research show the necessity of introducing differential diagnostics for HPABH_4_ into neonatal screening practice.

## Introduction

Hyperphenylalaninemias (HPA) are hereditary autosomal recessive disorders caused by defects in the hepatic phenylalanine hydroxylase (PAH) system. The prevalent HPA type is phenylketonuria (PKU) or PAH-deficiency (OMIM #261600) caused by mutations in the *PAH* gene which codes PAH. PKU accounts for approximately 98% of all HPA cases [1]. Its clinical feature is impaired postnatal cognitive development of varying severity as well as eczematous rash, autism, seizures, motor deficits, aberrant behaviour, and psychiatric symptoms [2]. The classical approach to PKU treatment is diet therapy which limits phenylalanine (Phe) consumption. However, this approach may significantly worsen the patient’s quality of life. Adult PKU patients stop following the recommendations on diet therapy in many cases which leads to depression, attention deficit disorders, aggressive behavior, and, as a result, desocialization. Resently, a number of alternative methods of PKU treatment have appeared. Sapropterin is a pharmacological analogue of tetrahydrobiopterin (BH_4_), a cofactor and a chaperone for PAH, can partially restore residual PAH activity in patients with so-called «mild» *РАН* mutations [3]. Pegvaliase enzyme replacement therapy lowers Phe levels in the adult patient’s blood regardless of their genotype but may show side effects [4]. Technologies using probiotic bacteria modified to process Phe from the patient’s intestines, are being developed [5]. There are also researches on alternative treatment methods using large neutral amino acids which competitively inhibit Phe transportation through the blood–brain barrier [6].

The HPA group also includes tetrahydrobiopterin-deficient hyperphenylalaninemias (HPABH_4_) caused by defects in genes, which code proteins that participate in BH_4_ synthesis and metabolism. BH_4_ is a cofactor for two other hydroxylases apart from PAH—tyrosine hydroxylase and tryptophan hydroxylase. Therefore, HPABH_4_ clinical findings include not only increased blood Phe levels, but also progressing cognitive and motor disturbances due to impaired syntheses of dopamine and serotonin. Phe restricted diet as a monotherapy is ineffective for HPABH_4_ patients as it does not solve the problem of neurotransmitter deficit. The most effective method for those patients is BH_4_ replacement therapy combined with levodopa and 5-hydroxytryptophan [7–9]. Other treatment options are folinic acid, melatonin, and dopamine agonists [7]. The most common disorder in this group is HPABH_4_A caused by mutations in the *PTS* gene: it accounts for 65.3% of all HPABH_4_ cases in Europe which in turn make around 2% of all HPA cases. HPABH_4_C caused by mutations in the *QDPR* gene, accounts for 24.9% of all HPABH_4_ in Europe [10].

The list of HPA disorders is presented in Table 1. Aside from PKU caused by mutations in the *PAH* gene, and HPABH_4_, related to tetrahydrobiopterin metabolism, HPAs also include DOPA-responsive dystonia and non-BH_4_-deficient hyperphenylalaninemia. DOPA-responsive dystonia is caused by deficit of sepiapterin reductase which participates in the last stage of tetrahydrobiopterin synthesis. Phe levels in blood do not increase because the absence of *SPR* expression activate alternative metabolic pathways which compensate for the lack of BH_4_. However, these compensatory mechanisms are partially missing in the brain causing central BH_4_ synthesis to decrease. The increase of Phe levels in the brain results in neurological symptoms: 7,8-dihydrobiopterin accumulation competitively inhibits tyrosine- and tryptophan hydroxylase causing a decrease in concentration of neurotransmitters [11, 12]. Thus, DOPA-responsive dystonia has clinical symptoms and pathogenesis similar to HPABH_4_ without actual HPA. Non-BH_4_-deficient hyperphenylalaninemia (HPANBH_4_) is the least studied form because the connection between mutations in the *DNAJC12* gene and HPA was first shown in 2017 [13–15]. The DnaJ homolog subfamily C member 12 protein coded by the *DNAJC12* gene, has a function similar to BH_4_: it is a chaperone for enzymes participating in neurotransmitter metabolism, including PAH. Thus, a defect in the *DNAJC12* gene leads to a phenotype similar to HPABH_4_ that is also responsive to BH_4_ therapy. Functions of DNAJC12 protein, treatment strategies and possible interactions with different *PAH* gene variants are studied by many scientific groups nowadays [16, 17].

**Table 1:** Hyperphenylalaninemia classification.

Disorder	OMIM	Gene	Protein
Phenylketonuria (PKU)	#261600	*PAH*	Phenylalanine hydroxylase (PAH)
Tetrahydrobiopterin-deficient hyperphenylalaninemia A (HPABH_4_A)	#261640	*PTS*	6-pyruvoyl-tetrahydropterin synthase (PTPS)
Tetrahydrobiopterin-deficient hyperphenylalaninemia B (HPABH_4_B)	#233910	*GCH1*	GTP cyclohydrolase I (GTPCH-1)
Tetrahydrobiopterin-deficient hyperphenylalaninemia C (HPABH_4_C)	#261630	*QDPR*	Dihydropterin reductase (DHPR)
Tetrahydrobiopterin-deficient hyperphenylalaninemia D (HPABH_4_D)	#264070	*PCBD*	Pterin-4-α-carbinolamine dehydratase I (PCD)
DOPA-responsive dystonia	#612716	*SPR*	Sepiapterin reductase (SR)
Non-tetrahydrobiopterin-deficient hyperphenylalaninemia (HPANBH_4_)	#617384	*DNAJC12*	J domain-containing protein 1 (JDP1, DNAJC12)

Hyperphenylalaninemia is detected via neonatal screening with analysis of Phe concentration in dried blood spots. At this timepoint the differential diagnostics between PKU and HPABH_4_ is not performed in Russia nowadays. After re-testing to confirm HPA, patients are observed by a geneticist and treated conservatively (diet therapy). Differentiating PAH-deficiency from the HPABH_4_ is a crucial task, seeing as the Phe restricted diet as a monotherapy is not suitable for patients with rare HPA forms. Without differential diagnostics these patients often receive the Phe restricted diet as the only treatment. At first, it is effective and lowers Phe levels, but long term it can lead to acute conditions caused by neurotransmitter deficit [18]. Blood Phe levels in patients with rare HPA types may vary from “mild HPA” to “classic PKU” diagnosis. It is impossible to differentiate a HPABH_4_ from *PAH*-deficiency by Phe levels in blood [19]. Some patients with HPABH_4_B can have normal Phe concentrations in the neonatal period [20]; DOPA-responsive dystonia is not characterized by blood Phe level increase [11]–these HPA forms may be missed during neonatal screening. The following methods could differentiate HPA forms in newborns: DHPR protein activity (HPABH_4_C) can be detected in dried blood spots; HPABH_4_A, -B and -C could be identified with pterin analysis in dried blood spots or urine [18, 21]. As an alternative or simultaneously, one could use a BH_4_ loading test which can identify BH_4_-responsive patients (all rare HPA forms) as well as patients with BH_4_-responsive *PAH*-deficiency.

Differential diagnostics should be included into the routine neonatal screening practice [9]. Pterin profile analysis became available in 2018 in Moscow and is carried out for the patients’ urine samples on individual request. Among patients with a PKU or HPA diagnosis, rare HPA types are detected by DNA diagnostics, including the Next generation sequencing (NGS) panel method [22]. In this study we present comprehensive data on Russian patients with rare HPA forms.

## Materials and methods

### Patients

Blood samples of 30 non-related patients with BH_4_-deficient HPA as well as 20 healthy relatives of the probands were analyzed. The written informed consents from the patients or their parents were obtained. The study was approved by the local ethics committee of the Research Centre for Medical Genetics (the approval number 2020-1/2).

### Methods

The research is retrospective and sums up the data of patients with BH_4_-responsive HPA accumulated in the DNA-diagnostic Laboratory of Research Centre for Medical Genetics. In this study we use various molecular genetic diagnostic methods and approaches.

#### DNA extraction

Genomic DNA was extracted from peripheral blood leukocytes using the WizardGenomic DNA Purification Kit (Promega, USA) according to the manufacturer’s protocol.

#### MLPA analysis

Multiplex ligation-dependent probe amplification (MLPA) which allows to detect common point substitutions in the *PTS* gene, was carried out using a programmable MC2 thermocycler (DNA-technology, Russia) in two stages. During the first stage, original oligonucleotides were annealed with the examined denatured DNA in the presence of thermostable DNA ligase for 1 hour. 5 μl of the reagent mixture contained: 10–50 ng genomic DNA, 0.16–10 fmol/μl of each oligonucleotide (Evrogen, Russia), 0.4 U Pfu DNA ligase (Helicon, Russia), ligation buffer (20 mmol Tris-HCl pH 7.5, 20 mmol KCl, 10 mmol MgCl_2_, 0.1% Igepal, 0.01 mmol rATP, 1 mmol DTT); 20–30 μl mineral oil. The second stage was standard PCR using oligoprimers complementary to sequence regions specifically synthesized in oligonucleotides. 15 μl of PCR reagent mixture was added into the mix, where a ligase reaction had been carried out. The mixture consisted of 0.25 μmol of each original oligoprimer (Evrogen, Russia), 200 μmol of each nucleoside triphosphate (Helicon, Russia), 1.0 U Biotaq DNA polymerase (BioMaster, Russia), PCR buffer (67 mmol Tris-HCl, 16.6 mmol (NH_4_)_2_SO_4_, 0.01% Twin-20; рН 8.8). The results were analyzed via vertical electrophoresis (20х20 cm) in 9% polyacrylamide gel, later stained with ethidium bromide and registered using GelDoc (BIO-RAD, USA) in UV lighting with 312 nm wavelength.

#### Quantitative MLPA analysis

To find deletions in the *PTS* gene, the point mutation detection system was expanded with sequences of *SIRT3*, *USP3*, and *B2M* reference genes, which always occur in the human genome in two copies. PCR following the ligase reaction was carried out using a FAM-labelled primer, which allows to detect results with fragment analysis on an ABI Prism 3100 analyzer (Applied Biosystems, USA). Mathematical estimations of the results were carried out with Coffalyser V8 (MRC-Holland, Netherlands).

#### Relationship confirmation

Genotyping was carried out using PCR with the «AmpFISTR Identifiler Direct PCR Amplification Kit» (Applied Biosystems, USA) for direct amplification of 16 human DNA loci according to the manufacturer’s protocol. The amplification products were separated on an ABI Prism 3100 analyzer (Applied Biosystems, USA).

#### Sanger sequencing

*PTS* and *QDPR* nucleotide sequences were identified with direct automated Sanger sequencing of the PCR product using forward and reverse primers. Fragments obtained via PCR amplification were used as a sequencing matrix. Sequencing was carried out on an ABI Prism 3100 analyzer (Applied Biosystems, USA) according to the manufacturer’s protocol.

The proband’s relatives were scanned for detected variants with Sanger sequencing of the target exons.

#### Next generation sequencing

NGS was carried out with a custom «PKU» AmpliSeq™ panel, which covers coding sequences and exon-intron junctions of *PAH*, *PTS*, *GCH1*, *PCBD1*, *QDPR*, *SPR*, and *DNAJC12* genes, using the manufacturer’s software. The panel includes two PCR primer pools, 68 primers total, with average amplicon length 158 bp. Library preparation and sequencing was carried out on Ion S5™ according to the manufacturer’s protocol. Sequencing data was analyzed using a standard automated algorithm offered by Termo Fisher Scientific (Torrent Suite™) as well as Gene-Talk software. Gene coverage: *PAH—*100%, *PTS—*98%, *GCH1–*87.2%, *PCBD1–*94%, *QDPR—*100%, *SPR–* 82.3%, *DNAJC12–*100%.

#### Data interpretation

In cases where the variant was described in no less than two literature sources as the cause of a disorder, it was classified as pathogenic. If it was not described previously or described only in one literature source, clinical significance (pathogenicity) was evaluated according to the NGS data interpretation recommendations [23].

To apply the PS4 criterion (variant prevalence in the affected cohort is much higher than that in the healthy cohort) we calculated the probability ratio. For a reference group, we used a custom database containing exome sequencing data of 1036 Russian patients from the DNA-diagnostic laboratory of Research Centre for Medical Genetics. The criterion was applied in those cases where the allele occurred in the patient cohort more than once. Probability ratio calculation for a single variant occurrence is inaccurate.

The following algorithms were used as *in silico* predictors: DANN, DEOGEN2, EIGEN, FATHMM-MKL, M-CAP, MVP, MutationAssessor, PrimateAI, REVEL, SIFT, MutationTaster.

#### Urinary pterin spectrum analysis

Pterin spectrum analysis for urine samples was carried out using high-performance liquid chromatography (HPLC). Control reagents: 6-neopterin (6-Neo), 7,8-dihydrobiopterin (BH_2_), pterin (Pte), 6-biopterin (6-Bio), 7-biopterin (7-Bio), L-(5-15N)-biopterin (IS-Bio), (5-15N)-7,8-dihydrobiopterin (IS-BH_2_) and D-(5-15N)-neopterin (IS-Neo), produced by Schircks Laboratories (Jona, Switzerland).

Urine samples of the control group and the HPA patients were oxidized using dithiothreitol. Oxidized samples were diluted with deionized water according to creatinine concentration (up to 0.1 g/l).

Before mass spectrometry the samples were diluted 10 times with mobile phase containing internal standards in concentrations 20, 20, 5 ng/ml—IS-BH_2_, IS-Neo, IS-Bio respectively.

Chromatographic separation was carried out on a Waters XBridge BEH Amide column (130Å, 3.5 μm, 2.1 mmX100mm) with a Waters XBridge BEH Amide precolumn (130Å, 3.5 μm, 2.1 mmX5mm). Mobile phase А – 20 mmol ammonium formate + 0.1% formic acid, mobile phase В –acetonitrile, flow rate– 0.4 ml/min. Gradient program: 0 minutes—6% А, 15 minutes 15% А, 17 minutes 15% А, 17.1 minutes 6% А and 3 minutes for column recovery.

The samples were analyzed using a Nexera HPLC system (Shimadzu, Japan) and a Q-Trap 5500® mass spectrometer (AB/SCIEX, Canada), curtain gas (CUR)– 20 psi, ion spray voltage (IS)– 5500 V, ion source heater temperature (TEM) - 600°C, source gas 1 (GS1)– 50 psi, source gas 1 (GS2)– 65 psi. Experimental MRM (Multiple reaction monitoring) transition parameters were optimized in positive ionization mode for each examined substance (Table 2), declustering potential (DP)– 120 V, entrance potential (EP)– 8 V, cell exit potential (CXP)– 15 V.

**Table 2:** MRM transition parameters.

Сompound	MRM	Collision energy (СЕ)
6-neopterin	254.1 >206.1	21
7,8-dihydrobiopterin	240.1 >165.1	27
pterin	164.1 >119.1	27
6-biopterin	238.1 >220.1	22
L-(5-15N)-biopterin	239.1 >221.1	22
(5-15N)-7,8-dihydrobiopterin	241.1 >166.1	27
D-(5-15N)-neopterin	255.1 >207.1	21

## Results

### HPABH_4_A, *PTS* gene

25 probands out of 30 had 2 variants in the *PTS* gene (50 chromosomes with *PTS* variants). In total, 18 different variants were detected in the *PTS* gene (Table 3) out of which only 4 were previously described in two or more literature sources as pathogenic: IVS1-3C>G, p.Asn52Ser, p.Pro87Ser, p.Thr106Met.

**Table 3:** Variants detected in the *PTS* gene (NM_000317.2).

Position in cDNA	Position in protein	Number of chromosomes	Prevalence, %	Pathogenicity criteria^a^	Variant description	Variant pathogenicity^b^
c.317C>T	p.Thr106Met	16	32.0		[24, 25]	P
c.216T>A	p.Asn72Lys	10	20.0	PS4, PM2, PM3, PP2, PP3, PP4	[26]	P
c.26G>A	p.Arg9His	4	8.0	PS4, PM2, PM3, PM5, PP2, PP4	[27]	P
c.94A>G	p.Ser32Gly	3	6.0	PS4, PM2, PM3, PP2, PP3, PP4	novel^c^	P
c.315-1G>A	IVS5-1G>A	2	4.0	PVS1, PS4, PM2, PP2, PP3, PP4	novel	P
c.335T>C	p.Val112Ala	2	4.0	PS4, PM2, PP2, PP3, PP4	novel	LP
c.187A>G	p.Ile63Val	2	4.0	PS4, PM2, PP2, PP3, PP4	[28]	LP
c.84-3C>G	IVS1-3C>G	1	2.0		[29, 30]	P
c.178T>G	p.Val59Gly	1	2.0	PM2, PP2, PP3, PP4	novel	VUS
c.400G>A	p.Glu134Lys	1	2.0	PM2, PM5, PP2, PP3, PP4	[30]	LP
c.158A>G	p.Tyr53Cys	1	2.0	PM2, PP2, PP3, PP4	novel	VUS
c.191A>T	p.Asp64Val	1	2.0	PM2, PP2, PP3, PP4	novel	VUS
c.155A>G	p.Asn52Ser	1	2.0		[24, 30]	P
c.259C>T	p.Pro87Ser	1	2.0		[30, 31]	P
c.35C>G	p.Ala12Gly	1	2.0	PM2, PM3, PP2, PP3, PP4	[32]	LP
c.108C>G	p.Asn36Lys	1	2.0	PM2, PP2, PP3, PP4	[33]	VUS
c.146A>G	p.His49Arg	1	2.0	PM1, PM2, PM3, PP2, PP3, PP4	[34]	LP
c.(?_26)_(216_?)		1	2.0	PVS1, PM2, PM3, PP4	novel	P
Total		50	100			

^a^Pathogenicity criteria abbreviations (PVS, PS, PM, PP) are given in accordance to the data interpretation recommendations.

^b^P–pathogenic variant, LP–likely pathogenic variant, VUS–variant with unknown clinical significance.

^с^Novel–variant, not previously described in literature.

7 variants were not previously described: p.Ser32Gly, p.Tyr53Cys, p.Val59Gly, p.Asp64Val, IVS5-1G>A, p.Val112Ala, c.(?_26)_(216_?). 7 more variants were described once, which does not allow to explicitly characterize them as pathogenic: p.Ala12Gly, p.Asn36Lys, p.His49Arg, p.Ile63Val, p.Asn72Lys, p.Glu134Lys. Classification of 14 variants using pathogenicity criteria allowed to characterize 5 of them as pathogenic (P), 5—as likely pathogenic (LP), 4 –as variants with unknown clinical significance (VUS); pathogenicity criteria for the detected variants were presented in Table 3. The variants with unknown clinical significance might, nevertheless, be attributed to the HPABH_4_A phenotype if additional confirmation is obtained.

Variants p.Thr106Met (32%), p.Asn72Lys (20%), p.Arg9His (8%), and p.Ser32Gly (6%) are the most common pathogenic *PTS* variants in Russian patients.

Among 25 families with detected *PTS* variants (Table 4) 10 had material of both parents available which allowed to confirm the variants’ trans position (families № 3, 4, 6, 11, 12, 13, 17, 20, 21, 22). In one family maternal carriage was confirmed seeing as the father’s material was unavailable (№ 25).

**Table 4:** Genotypes of patients with detected variants in the *PTS* gene (NM_000317.2), their family examination and pterin spectrum analysis results.

Family №	*PTS* allele 1	*PTS* allele 2	Method	Family analysis	Pterins
1	c.216T>A	c.317C>T	*PTS* sequencing ^c^	- ^a^	-
2	c.84-3C>G	c.315-1G>A	*PTS* sequencing	-	-
3	c.94A>G	c.178T>G	*PTS* sequencing	Carriage confirmed in the mother and the father	-
4	c.216T>A	c.317C>T	*PTS* sequencing	Carriage confirmed in the mother and the father	-
5	c.94A>G	c.317C>T	*PTS* sequencing	-	-
6	c.26G>A	c.317C>T	*PTS* MLPA, *PTS* sequencing	Carriage confirmed in the mother and the father, diagnosis confirmed in the brother	-
7	c.317C>T	c.400G>A	*PTS* sequencing	-	+^b^
8	c.317C>T	c.317C>T	*PTS* MLPA, *PTS* sequencing	-	-
9	c.158A>G	c.216T>A	*PTS* sequencing	-	-
10	c.335T>C	c.335T>C	*PTS* sequencing	-	-
11	c.317C>T	c.216T>A	*PTS* MLPA, *PTS* sequencing	Carriage confirmed in the mother and the father	-
12	c.216T>A	c.317C>T	*PTS* MLPA, *PTS* sequencing	Carriage confirmed in the mother and the father	-
13	c.216T>A	c.317C>T	*PTS* sequencing	Carriage confirmed in the mother and the father	-
14	c.191A>T	c.317C>T	*PTS* sequencing	-	-
15	c.26G>A	c.317C>T	*PTS* sequencing	-	-
16	c.155A>G	c.259C>T	*PTS* sequencing	-	-
17	c.35C>G	c.216T>A	NGS panel ^d^, *PTS* sequencing	Carriage confirmed in the mother and the father	+
18	c.216T>A	c.317C>T	NGS panel	-	+
19	c.26G>A	c.108C>G	NGS panel	-	+
20	c.146A>G	c.216T>A	NGS panel, *PTS* sequencing	Carriage confirmed in the mother and the father	+
21	c.94A>G	c.317C>T	NGS panel, *PTS* sequencing	Carriage confirmed in the mother and the father	+
22	c.26G>A	c.(?_26)_(216_?)	NGS panel, *PTS* sequencing, quantitative MLPA analysis	Carriage confirmed in the mother and the father	+
23	c.216T>A	c.317C>T	NGS panel	-	+
24	c.187A>G	c.315-1G>A	*PTS* sequencing	-	+
25	c.187A>G	c.317C>T	NGS panel, *PTS* sequencing	Carriage confirmed in the mother only	+

^a^«-» - analysis not carried out.

^b^«+» - detected urinary pterin spectrum shifts corresponding to 6-pyruvoyltetrahydropterin synthase defect.

^c^Sequencing—direct automated Sanger sequencing.

^d^NGS panel—next generation sequencing using a custom “PKU” panel including *PAH*, *PTS*, *GCH1*, *PCBD1*, *QDPR*, *SPR* and *DNAJC12* genes.

Probands № 18–22 were previously described by our group, *Kuznetcova et al* [22]. This study presents updated information on these families, with family examination and biochemical analysis.

Proband № 22, as confirmed by sequencing with a «PKU» NGS panel and subsequent Sanger sequencing, had the variant in homo-/hemizygous state. During the family analysis the p.His49Arg variant was detected in the mother in heterozygous state and not detected in the father. After the relation of both parents was confirmed a gross deletion in the *PTS* gene was suspected in the proband and his father. MLPA with existing oligoprimers for *PTS* variant detection was modified. As a result of quantitative analysis the deletion of exons 1, 2, 4, 6 of the *PTS* gene (other exons were not investigated) was detected in heterozygous state both in the proband and the father. Deletion boundaries were not identified, nomenclature was given using the terminal oligoprimers’ coordinates: c.(?_26)_(216_?). The deletion probably affects the entire *PTS* gene.

Urinary pterin spectrum analysis was carried out for 10 probands with detected *PTS* variants (Table 5). All patients had a decrease in pterin (Pte) concentration. 8-biopterin (Bio) concentration was low in 8 patients (except № 7 and 17). 6-neopterin (Neo) concentration was above average in 8 patients (except № 24 and 25). Also most patients (except № 7, 20 and 24) were shown to have decreased 7,8-dihydrobiopterin (BH_2_) levels. Neo/Bio ratio was high and Bio/(Neo+Bio) low in all examined patients.

**Table 5:** Biochemical analysis of urinary pterin spectrum for patients with detected *PTS* variants (NM_000317.2).

Family №	BH_2_^b^	Neo^c^	Pte^d^	Bio^e^	Neo/Bio	Bio/ (Neo+Bio) (%)
7	**13.80**^**a**^	6.16	0.10	**0.28**	22,0	4,35
17	4.26	4.49	0.04	**0.25**	18,0	5,27
18	4.65	5.16	0.02	0.02	258,0	0,39
19	4.73	4.75	0.06	0.19	25,0	3,85
20	**10.10**	5.69	0.09	0.06	94,8	1,04
21	1.03	7.03	0.06	0.12	58,6	1,68
22	3.68	6.04	0.03	0.07	86,3	1,15
23	1.32	6.94	0.01	0.04	173,5	0,57
24	**10.13**	**3.35**	0.04	0.07	47,9	2,05
25	0.43	**2.19**	0.02	0.10	21,9	4,37
Reference values, mmol/mol of creatinine	5–15	0.5–3.5	0.1–1.6	0.2–3.9	0.5–15	10–70

^**a**^**Bold**–values inside the reference limits.

^b^BH_2_−7,8-dihydrobiopterin.

^c^Neo—6-neopterin.

^d^Pte—pterin.

^e^Bio—6-biopterin.

### HPABH_4_C, *QDPR* gene

4 out of 30 examined probands had 2 variants in the *QDPR* gene (8 mutant chromosomes in total). There were 5 different variants, 2 of which were previously described in literature as pathogenic: p.Ser115Leu, p.Tyr150Cys; 3 were novel: p.Arg31Trp, p.Ala69Pro, p.Ala135Asp. According to the pathogenicity prediction criteria two of those were classified as “likely pathogenic”, one–as “unknown clinical significance”. This last variant (p.Ala69Pro) could be attributed to the HPABH_4_C phenotype with additional confirmation. The detected variants’ pathogenicity is presented in Table 6.

**Table 6:** *QDPR* variants (NM_000320.2).

Position in cDNA	Position in protein	Number of chromosomes	Prevalence, %	Pathogenicity criteria^a^	Variant description	Variant pathogenicity^b^
c.91C>T	p.Arg31Trp	2	25.0	PS4, PM2, PP2, PP3, PP4	Novel^c^	LP
c.404C>A	p.Ala135Asp	2	25.0	PS4, PM2, PP2, PP3,PP4	novel	LP
c.449A>G	p.Tyr150Cys	2	25.0		[30, 35]	P
c.205G>C	p.Ala69Pro	1	12.5	PM2, PP2, PP3, PP4	novel	VUS
c.344C>T	p.Ser115Leu	1	12.5			P
Total		8	100			

^a^Pathogenicity criteria abbreviations (PVS, PS, PM, PP) are given in accordance to the data interpretation recommendations.

^b^P–pathogenic variant, LP–likely pathogenic variant, VUS–variant with unknown clinical significance.

^c^Novel–variant, not previously described in literature.

Family analysis was carried out for 2 of 4 families with HPABH_4_C; both parents were confirmed to be heterozygous carriers (Table 7). In this case, the family analysis did not increase the variants’ pathogenicity due to the impossibility of PM3 criterion application. Nevertheless, we confirmed the variants’ homozygosity in probands № 28 and 29. In family № 26 only the mother was confirmed to be a heterozygous carrier due to unavailability of the father’s material. Family analysis was not carried out for family № 27 due to unavailability of material of either parent. Pterin spectrum analysis for HPABH_4_C patients shown in the Table 7 confirms that this kind of analysis is unspecific for this HPA form [7].

**Table 7:** Genotypes of patients with detected variants in the *QDPR* gene (NM_000320.2), family examination and biochemical pterin spectrum analysis results.

Family №	*QDPR* allele 1	*QDPR* allele 2	Method	Family examination	Urine pterin spectrum analysis
26	c.404C>A	c.404C>A	*QDPR* sequencing ^b^	Carriage confirmed in the mother	Uncharacteristic changes
27	c.205G>C	c.344C>T	*QDPR* sequencing	- ^a^	-
28	c.91C>T	c.91C>T	*QDPR* sequencing	Carriage confirmed in the mother and the father	-
29	c.449A>G	c.449A>G	*QDPR* sequencing	Carriage confirmed in the mother and the father	normal

^a^ «-» - analysis not carried out.

^b^Sequencing—direct automated Sanger sequencing.

### DOPA-responsive dystonia, *SPR* gene

Patient № 30 had no clinical evidence of HPA, however, according to her parents, exome sequencing (results unavailable) showed variants in the *SPR* gene. Sequencing with a custom “PKU” NGS panel detected a с.524С>А (p.Ala175Asp) variant in the *SPR* gene (NM_003124.4) in homo-/hemizygous state. Family examination and pterin spectrum analysis could not be carried out. According to the pathogenicity evaluation criteria (PM2, PP3) this variant can be classified as “unknown clinical significance”. The patient receives BH_4_ pharmacological analogues with a positive effect. This might indicate the disorder is caused by a defect in one of the genes responsible for BH_4_ synthesis and metabolism, and therefore the с.524С>А (p.Ala175Asp) *SPR* variant (NM_003124.4) could be pathogenic.

## Discussion

### HPABH4 prevalence

The DNA-diagnostic laboratory of Research Centre for Medical Genetics has accumulated multiple years’ data of routine diagnostics for patients with hereditary disorders. The full cohort of PKU and HPA patients consists of 3452 non-related probands. HPABH_4_ accounts for approximately 0.87% of all HPA cases in Russian Federation (30 out of 3452). However, the laboratory has incomplete data of *PAH*-deficiency patients, since small quantity regions detect frequent mutations in the *PAH* gene and patients with the most common *PAH* variants are not included in the mentioned cohort. On the other hand, the most complicated cases are often referred to our laboratory because it has the full range of diagnostic methods. Thus, the prevalence of HPABH_4_ (0.87%) is probably overvalued. Really it accounts for around 0.5%, as shown before by *Kuznetcova et al* [22].

The clinical case of DOPA-responsive dystonia was taken into account for HPABH_4_ prevalence evaluation. However, as Phe levels are not increased, these patients are much less detectable. Also, patients with DOPA-responsive dystonia are not initially included in the PKU and HPA cohort because they are overlooked during neonatal screening.

A more accurate evaluation of HPABH_4_ prevalence would be possible only if differential HPABH_4_ diagnostics were introduced to neonatal screening practice.

Global HPABH_4_ prevalence varies greatly. On average, in Europe it is 1.5–2% [10]. According to a research conducted in Spain, it is 8.4% [28] or 5% [16]. In Saudi Arabia it is 25% of all HPAs [36]. In Brazil, it accounts for 1.71% [37]. A research in China shows that 20.5% of all HPAs are BH_4_-deficient. More diverse data is currently unavailable, possibly due to the absence of differential HPABH_4_ and PKU diagnostics. We can assume that HPABH_4_ is less prevalent in Russia than in European countries.

### HPABH4 structure in Russian Federation

HPABH_4_A prevalence in this study was 83.3% of all HPABH_4_ cases (25 out of 30), HPABH_4_C – 13.3% (4 cases out of 30). The most common HPABH_4_ is HPABH_4_A, which occurs in Russia more frequently than on average in European countries (Europe—65.3% HPABH_4_A, 24.9% HPABH_4_C) [10].

An HPABH_4_A shift has been noted in China as well, where its prevalence is 96.2%, HPABH_4_C – 3.4%, HPABH_4_B – 0.5%, no other rare HPA types described [38]. In Saudi Arabia, only HPABH_4_A was noted [36]. In Spain, HPABH_4_A and–C account for 38.1% each, HPABH_4_B – 25% [28].

This high HPABH_4_A percentage in Russian residents is probably caused by accumulation of the same *PTS* variants: p.Thr106Met, p.Asn72Lys, p.Arg9His, p.Ser32Gly, IVS5-1G>A, p.Val112Ala, p.Ile63Val. The first three account for 30 out of 50 chromosomes with detected *PTS* variants (60%).

This study presents a single case of DOPA-responsive dystonia. There is still no data available on other rare HPA forms in Russia. At present, probands without detected *PAH* variants have an opportunity of full HPA-related gene spectrum analysis using an NGS panel (*PTS*, *GCH1*, *PCBD1*, *QDPR*, *SPR*, *DNAJC12*). However, no patients with mutations in *GCH1* (autosomal recessive form), *PCBD1* or *DNAJC12* genes are currently identified.

### Differential HPA diagnostics

Out of 29 probands with confirmed HPABH_4_A and HPABH_4_C, 28 were detected by DNA diagnostics, 24 were initially referred to the laboratory with a PKU diagnosis. Before the implementation of the “PKU” NGS panel, patients underwent a complex diagnostic procedure, including a screening for most common *PAH* variants, *PAH* Sanger sequencing, MLPA allowing to detect gross rearrangements in the *PAH* gene, and Sanger sequencing of *PTS* and *QDPR*, which was expensive and time-consuming. Most probands were diagnosed with HPABH_4_ at puberty then the conservative diet was changed to sapropterin replacement therapy.

Proband № 24 had a screening for most common *PAH* variants simultaneously with a biochemical pterin spectrum analysis. This showed an absence of common *PAH* variants and a presence of biochemical changes typical for a defect in the *PTS* gene. Sanger sequencing of *PTS* confirmed the HPABH_4_A diagnosis.

Biochemical pterin spectrum analysis was carried out for patients № 2, 6, 18–23, 25, and 28 retrospectively to improve the method. At the moment, this procedure is not included in routine neonatal screening. Clinicians often send patients to pterin spectrum analysis if their Phe levels can be classified as “mild HPA” and HPABH_4_ can be suspected. This approach is incorrect, seeing as Phe levels are not indicative of HPABH_4_. This statement is confirmed by the present research, since 86% of the patients in the studied cohort initially had a PKU diagnosis. The situation shows the necessity of introducing differential diagnostic methods for HPABH_4_ and other HPA types into routine neonatal screening practice.

The DOPA-responsive dystonia case once again shows the difficulty of differential diagnostics for this particular disorder. Without a blood Phe level increase patients are not identified during neonatal screening and come to neurologists’ attention only when a serious clinical picture develops.

Determining *PTS* and *QDPR* mutation spectra makes differential HPABH_4_ molecular genetic diagnostics possible in Russian Federation. However, biochemical differential diagnostic methods are less expensive, less time-consuming, and simpler in execution. Its introduction into routine neonatal screening practice for early detection of HPABH_4_ patients should be Russian screening program’s priority.
